# Hemocyanin Modification of Chitosan Scaffolds with Calcium Phosphate Phases Increase the Osteoblast/Osteoclast Activity Ratio—A Co-Culture Study

**DOI:** 10.3390/molecules25194580

**Published:** 2020-10-07

**Authors:** Benjamin Kruppke, Christiane Heinemann, Jana Farack, Simy Weil, Eliahu David Aflalo, Amir Sagi, Thomas Hanke

**Affiliations:** 1 Max Bergmann Center of Biomaterials and Institute of Materials Science, Technische Universität Dresden, Budapester Str. 27, D-01069 Dresden, Germany; christiane.heinemann@tu-dresden.de (C.H.); jana.farack@gmail.com (J.F.); thomas.hanke@tu-dresden.de (T.H.); 2 Department of Life Sciences, Ben-Gurion University of the Negev, Beer-Sheva 8410501, Israel; simmi@bgu.ac.il (S.W.); aflaloe@bgu.ac.il (E.D.A.); sagia@bgu.ac.il (A.S.); 3 Department of Life Sciences, Achva Academic College, Arugot 7980400, Israel; 4 The National Institute for Biotechnology in the Negev, Ben-Gurion University of the Negev, Beer-Sheva 8410501, Israel

**Keywords:** bioactivity, brushite, co-culture, crayfish hemocyanin, hydroxyapatite, osteoblasts, osteoclasts

## Abstract

The ongoing research on biomaterials that support bone regeneration led to the quest for materials or material modifications that can actively influence the activity or balance of bone tissue cells. The bone biocompatibility of porous chitosan scaffolds was modified in the present study by the addition of calcium phosphates or hemocyanin. The first strategy comprised the incorporation of calcium phosphates into chitosan to create a biomimetic chitosan—mineral phase composite. The second strategy comprised dip-coating of chitosan scaffolds with hemocyanin extracted from crayfish hemolymph. The cytocompatibility was assessed in a mono-culture of human bone marrow stromal cells (hBMSCs) and their differentiation to osteoblasts; in a mono-culture of human monocytes (hMs) and their maturation to osteoclasts; and in a co-culture of hBMSC/osteoblasts—hM/osteoclasts. Mineral incorporation caused an increase in scaffold bioactivity, as shown by reduced calcium concentration in the cell culture medium, delayed differentiation of hBMSCs, and reduced osteoclastic maturation of hMs in mono-culture. Dip-coating with hemocyanin led to increased proliferation of hBMSCs and equivalent osteoclast maturation in mono-culture, while in co-culture, both an inhibitory effect of mineral incorporation on osteoblastogenesis and stimulatory effects of hemocyanin were observed. It was concluded that highly bioactive scaffolds (containing mineral phases) restrain osteoblast and osteoclast development, while hemocyanin coating significantly supports osteoblastogenesis. These influences on the osteoblasts/osteoclasts activity ratio may support scaffold-driven bone healing in the future.

## 1. Introduction

Human bone is a complex and dynamic system, and efforts to study any aspect of this system in vitro will thus require a framework that is also both complex and dynamic. To study the cytocompatibility of degradable bone replacement materials with respect to bone as host tissue, a variety of in vitro cell cultures have been tested, including osteoblast/osteoclast co-cultures. The use of this type of culture in studies on the influence of biomaterials on bone regeneration and their integration into the remodeling process of bone has been comprehensively reviewed by Zhu et al. [1]. Of particular relevance are co-culture models that do not require the addition of osteoclast-stimulating factors, such as RANKL (receptor activator of NF-κB ligand), and that may be cultivated directly on the biomaterials under investigation, as has been done by the authors of the present study [2,3]. One of the most important functions of bone substitute materials for human bone is the stimulation of bone-forming cells, namely, osteoblasts, derived from human bone marrow stromal cells (hBMSC). No less important are the bone-resorbing cells, namely, osteoclasts derived from human monocytes (hM), which provide and enhance non-inflammatory biomaterial resorption [4,5,6]. Importantly, in the context of biomaterial development, increased osteoclastic activity may result in indirect stimulation of osteoblasts via osteoblast/osteoclast cross-talk, which may ensure local bone regeneration as a consequence of biomaterial resorption.

The osteoblast/osteoclast activity ratio in human co-culture models can be affected by a number of factors, particularly the bioactivity of the introduced biomaterial [7] and extracellular calcium concentrations. The role of calcium cations—both those derived from composite materials containing calcium phosphate and those added to the cell culture medium—has been intensively investigated, as indicated by Matsui and Irie as well as Glenske et al. [8,9]. The bioactivity of a biomaterial that affects the extracellular calcium ion concentration and mineral precipitation from physiological solutions is regarded as a key factor in determining the osteoblast/osteoclast ratio during bone regeneration [10]. Calcium ions are fundamentally important for bone healing owing to their dual role as inorganic components of the bone composite material and as osteogenic factors. Extracellular calcium ion concentrations have been shown to stimulate osteoblastic activity up to a concentration of 8 mM [11,12] and to affect osteoclastic resorption [13].

In the present study, osteoblast/osteoclast co-cultures were used in an investigation of a biomaterial based on a chitosan scaffold modified with two different calcium phosphates as well as by the introduction of a crustacean-derived hemocyanin, which significantly enhanced the adhesion of hBMSCs to the scaffold [14]. The exoskeleton of crustaceans is composed mainly of chitin and is subject to cyclical assembly and disassembly in which various proteins, such as hemocyanin, play an important role [15]. In the crustacean species *Cherax quadricarinatus*, the red-claw crayfish, hemocyanin is found in the hemolymph, where it contributes mainly to oxygen transport [15] and to the immune response [16]. Recently, it was suggested that hemocyanin is also involved in sclerotization of the new exoskeleton after ecdysis [17]. During the molt cycle, hemocyanin (and other proteins) play an important role in the deposition of calcium carbonate (CaCO_3_)—either as crystalline calcite or as the stable form of amorphous CaCO_3_—in new cuticular chitinous structures [18,19]. The resulting naturally occurring composite material comprising chitin, hemocyanin, and calcium minerals might serve as a biomimetic approach for bone regeneration.

In the present study, we investigated the use of calcium phosphates to alter the scaffolds bioactivity, in terms of influencing the calcium ion concentration in the surrounding liquid, and used hemocyanin to improve the cytocompatibility of chitosan scaffolds with respect to osteoblast–osteoclast interaction. To this end, we used an in vitro co-culture model of osteoblasts and osteoclasts, derived from hBMSC and hM, respectively. To evaluate the biomaterials’ cytocompatibility, the influence of modifications to the biomaterials on the ratio of bone-forming to bone-resorbing cells cultivated directly on their surface was investigated. The differentiation of the cells according to their differentiation cascade was demonstrated by determination of alkaline phosphates (ALP) and tartrate-resistant phosphatase 5b (TRAP 5b), both specific for their cell type.

## 2. Results

### 2.1. Bioactivity and Mechanical Properties

**Figure 1:**
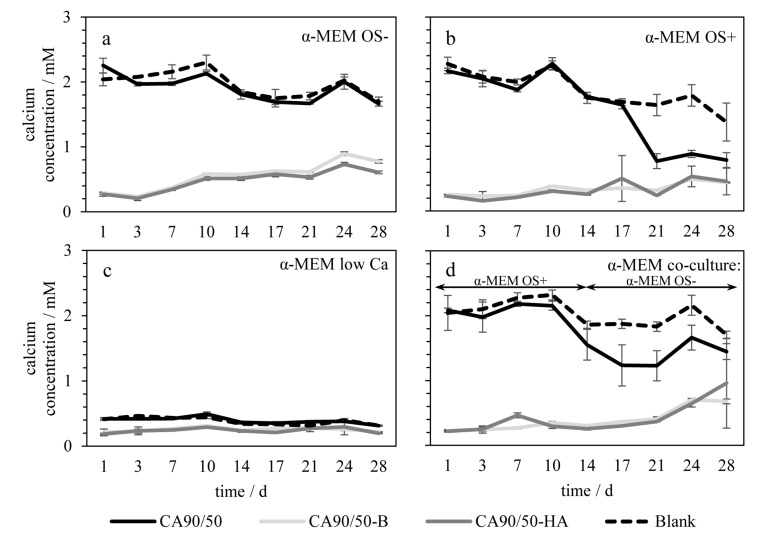
Calcium concentration in the cell culture media during incubation of scaffolds without cells: (**a**) without osteogenic supplements, (**b**) with osteogenic supplements, (**c**) with low initial calcium concentration, and (**d**) with co-culture supplemented regime. Incubation of pure and calcium-phosphate-modified chitosan scaffolds was performed.

During incubation in basal medium without osteogenic supplements, designated α-MEM OS-, chitosan scaffolds (designated CA90/50; see Section 4.1) modified with the calcium-phosphate-containing minerals, brushite CaHPO_4_·2H_2_O, or hydroxyapatite Ca_10_(PO_4_)_6_(OH)_2_ (designated CA90/50-B and CA90/50-HA, respectively), showed significantly different influences on the calcium ion concentration, which were related to the mineral content of the modified scaffolds. Pure chitosan scaffolds incubated in an α-MEM OS- medium did not reduce calcium concentrations in comparison to the values for the blank (plain CA90/50 scaffolds) (Figure 1a). In contrast, brushite-modified (CA90/50-B) and hydroxyapatite-modified (CA90/50-HA) chitosan scaffolds caused a drastic reduction in calcium concentration from the beginning of incubation (1 day: < 0.3 mM; Figure 1a), indicating their high bioactivity (compare supplementary scanning electron micrographs, Figure S1). During incubation of CA90/50-B and CA90/50-HA, repeated refreshment of medium led to a slow increase in calcium concentration to values that were still below 1 mM after 28 days (gray lines in Figure 1a). In contrast, the pure chitosan scaffolds (CA90/50) caused a drop of calcium concentration after 17 days and 14 days in α-MEM OS+ (Figure 1b) and α-MEM co-culture (Figure 1d), respectively. Surprisingly, even under conditions of reduced calcium concentration in the initial medium (α-MEM low Ca; Figure 1c), which might have favored brushite dissolution, both calcium-phosphate-modified scaffolds caused a drop of calcium concentration in the cell culture medium to values of about 0.2 mM in comparison to the initial value of 0.4 mM.

**Figure 2:**
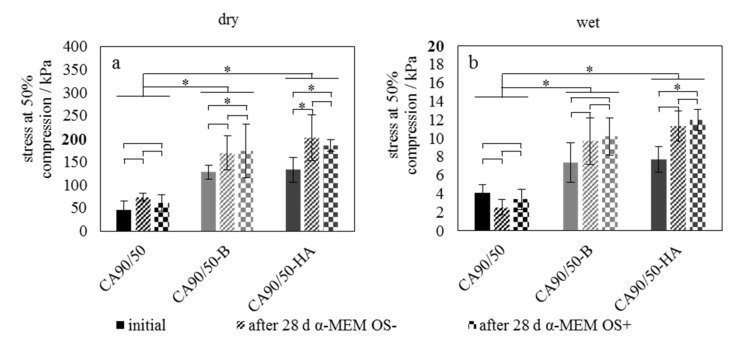
Stress at 50% uniaxial compression of pure and calcium-phosphate-modified chitosan scaffolds in (**a**) dry state and (**b**) wet state, both in the initial state after preparation of the scaffolds and after incubation in cell culture medium without and with osteogenic supplements over 28 days. An asterisk indicates significant difference with a *p* value of <0.05.

The mechanical strength of pure chitosan scaffolds in the dry state was significantly lower than that of the calcium-phosphate-modified scaffolds (Figure 2a). This effect was evident in the initial state as well as after incubation in the cell culture media. Interestingly, the incubation caused a slight increase in mechanical strength of samples in the dry state, which was not expected as rehydrated scaffolds tend to lose mechanical strength owing to degradation, causing lowered mechanical values when tested in both the dried and wet state. This increase in strength in the dried state after incubation was significant for CA90/50-HA initial vs. OS+ and initial vs. OS- as well as for CA90/50-B initial vs. OS+. Performing strength test in the wet state, the scaffolds showed significantly reduced mechanical stress at 50% compression compared to the dried scaffolds (Figure 2b). Once again, both calcium-phosphate-modified scaffolds exhibited significantly higher stress values compared to pure scaffolds. Enhanced mean values of CA90/50-B and CA90/50-HA (even though only significant for CA90/50-HA initial vs. OS+) were obtained after incubation in cell culture media.

### 2.2. Influence of Calcium Phosphates in Scaffolds on hBMSCs and hMs in Mono- and Co-Culture

**Figure 3:**
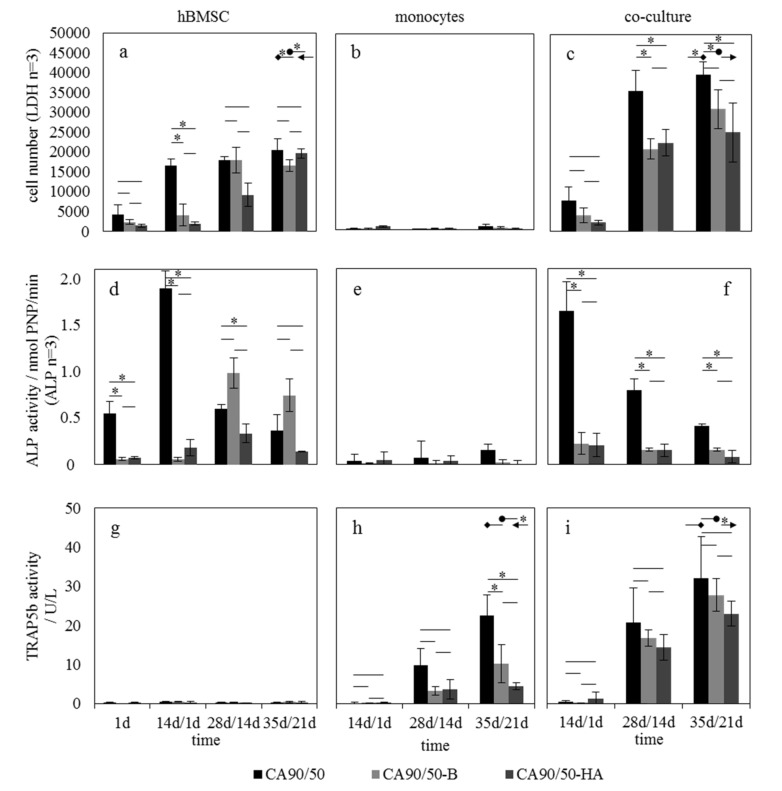
Cell culture analysis of chitosan scaffolds without and with brushite and hydroxyapatite modification. Adhesion and proliferation data are presented in terms of cell numbers (LDH) in (**a**) hBMSC mono-culture, (**b**) hM mono-culture, and (**c**) co-culture. Osteogenic differentiation of the cells was analyzed in terms of alkaline phosphates (ALP) activity (**d**–**f**), while osteoclastic ripening was measured in terms of TRAP5b activity (**g**)–(**i**). The arrow tails in (**a**,**c**) and those in (**h**,**i**) show the result of significance analysis for selected candidate pairs of values. An asterisk is placed at both arrow tails to indicate significant difference with a *p* value of <0.05.

The modification of chitosan scaffolds with brushite or hydroxyapatite influences their bioactivity. This, in turn, is believed to change the cellular reaction and ratio of osteoblasts to osteoclasts activity. In support of this notion, the culture of hBMSCs in mono-culture on the different chitosan scaffolds revealed a trend but not a significantly different adhesion, with the highest value being that for pure chitosan and the lowest value, for CA90/50-HA (Figure 3a). During hBMSC culture, this initial difference diminished and led to equal cell numbers after 35 days.

It can be seen that hMs have a significantly lower LDH activity than hBMSCs in mono-culture (Figure 3b). The LDH activity in co-culture is a combination of the LDH activity of osteoblasts and hM/osteoclasts. Therefore, the observed changes are based on the assumption that the low LDH activity of monocytes in mono-culture (which does not vary with time) is also present in co-culture. Thus, the change in LDH activity in co-culture is primarily attributed to the osteoblasts. In co-culture, a higher combined quantity of hBMSCs and hMs was measured on pure chitosan scaffolds than on brushite-modified and hydroxyapatite-modified scaffolds, which had significantly lowered cell numbers at 28 days/14 days (pure CA90/50 vs. CA90/50-B and CA90/50-HA) and 35 days/21 days (pure vs. CA90/50-HA) (Figure 3c). For the proliferation of cells in hBMSC mono-culture (Figure 3a) and co-culture (Figure 3c), there was a significant difference between the materials CA90/50 and CA90/50-B as well as between CA90/50 and CA90/50-HA but not between CA90/50-B and CA90/50-HA. Therefore, co-culture on CA90/50 and CA90/50-B showed significantly increased hBMSC proliferation in comparison to hBMSC mono-culture.

Osteogenic differentiation in terms of ALP activity of hBMSCs in mono-culture was highest after 14 days on CA90/50, as indicated by a significantly increased ALP maximum (Figure 3d). In comparison to CA90/50, calcium-phosphate-modified scaffolds showed lower ALP maxima, which were also shifted to later points in time (around 28 days). The ALP expression of the cells in hBMSC mono-culture (Figure 3d) showed a significant difference between all three materials, while in co-culture (Figure 3f), ALP expression was significantly different between CA90/50 and CA90/50-B and between CA90/50 and CA90/50-HA but not between CA90/50-B and CA90/50-HA. The hMs did not influence the ALP values (**∫**e). In co-culture, the ALP maximum was detected for all scaffolds on day 14 (Figure 3f), but ALP activity was significantly higher for osteoblasts on pure scaffold than on the modified scaffolds (CA90/50-B and CA90/50-HA).

As expected, the osteoclastic marker TRAP5b was not present in the hBMSC mono-culture (Figure 3g) but was found in hM mono- and co-culture for all scaffold types up to day 35. In hM mono-culture, significantly increased TRAP5b values were recorded for pure scaffolds vs. brushite- and hydroxyapatite-modified scaffolds (Figure 3h). The same trend (but not statistically significant) was found in co-culture. A significant difference in TRAP5b expression on day 35 was found only for a comparison of co-culture and hM culture for cells on CA90/50-HA (Figure 3h,i).

### 2.3. Influence of Hemocyanin-Modified Scaffolds on hBMSC and hM in Mono- and Co-Culture

**Figure 4:**
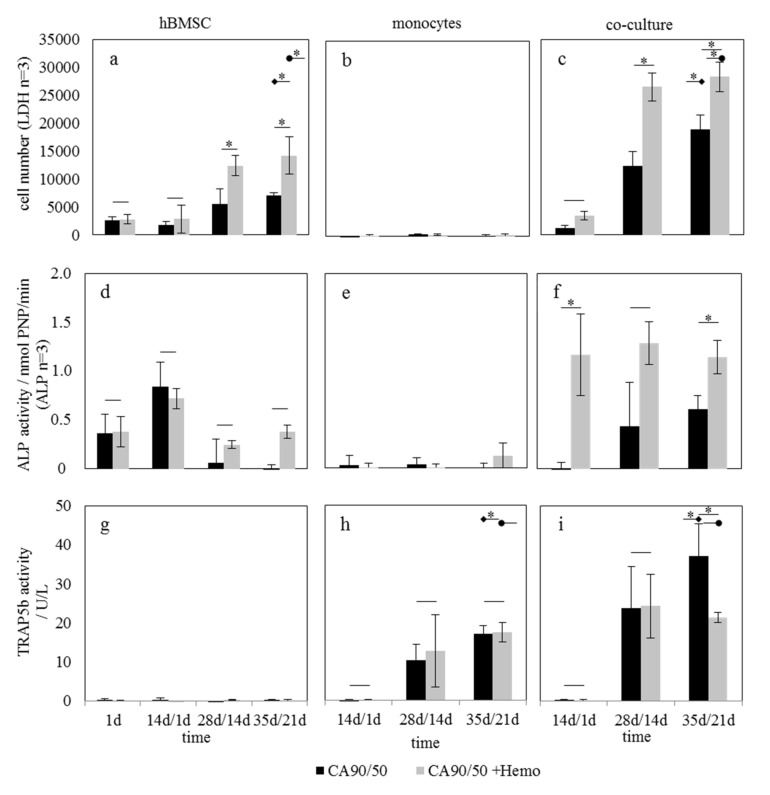
Cell culture analysis of chitosan scaffolds with and without hemocyanin modification. Adhesion and proliferation data are presented in terms of cell numbers (LDH) in (**a**) hBMSC mono-culture, (**b**) hM mono-culture, and (**c**) co-culture. Osteogenic differentiation of these cells was analyzed in terms of ALP activity (**d**–**f**), while osteoclastic ripening was measured in terms of TRAP5b activity (**g**–**i**). The arrow tails in (**a**,**c**) as well as those in (**h**,**i**) show the result of significance analysis for selected candidate pairs of values. An asterisk is placed on both arrow tails to indicate significant difference with a *p* value of <0.05.

Modification of chitosan scaffolds with hemocyanin resulted in equal adhesion of hBMSC compared to pure chitosan scaffolds and caused a significant enhancement of proliferation by day 35 (Figure 4a). For the proliferation of cells in hBMSC mono-culture (Figure 4a) and co-culture (Figure 4c), there was a significant difference between the materials CA90/50 and CA90/50+Hemo. In hM mono-culture (Figure 4b), low LDH activity was measured. Therefore, in co-culture the increase in cell number was mainly attributed to the hBMSC, as described above (Figure 4c). The LDH activity in co-culture was again significantly higher compared to mono-culture, even though there was no further addition of osteogenic supplements after addition of hMs on day 14 to the hBMSC pre-culture. Additionally, there was a significantly enhanced proliferation on hemocyanin-modified scaffolds.

Osteogenic differentiation of hBMSCs in mono-culture showed a maximum of ALP activity at day 14, which was independent of hemocyanin modification (Figure 4d). As expected, no ALP activity was detected in the hM mono-culture (Figure 4e), whereas in co-culture ALP, activity was higher and shifted to later points in time in comparison to the hBMSC mono-culture (Figure 4f). More importantly, ALP activity in co-culture was increased on the hemocyanin-modified scaffolds (CA90/50+Hemo). Thus, ALP expression of the cells in hBMSC mono-culture (Figure 4d) showed no significant difference between materials CA90/50 and CA90/50+Hemo but a significant difference in co-culture (Figure 4f).

As expected, the osteoclastic indicator TRAP5b was not found in hBMSC mono-culture (Figure 4g) but showed a constant increase in the hM culture irrespective of the scaffold type (Figure 4h). In co-culture, TRAP5b activity was increased on CA90/50 in comparison to hM culture (Figure 4h,i).

## 3. Discussion

### 3.1. Bioactivity and Mechanical Properties

From the calcium ion concentrations in the culture media, it can be clearly seen that the addition of both brushite and hydroxyapatite to the chitosan scaffolds caused a significant increase in bioactivity, which is determined here as the ability of the scaffold to induce precipitation of calcium phosphates from the medium, probably owing to the effect of brushite and hydroxyapatite as nucleation germs [20,21]. This did not occur with when pure chitosan scaffolds were placed in culture media. Nevertheless, pure chitosan scaffolds incubated in medium with osteogenic additives did show mineral formation, but not necessarily for the same reason as that presented above. This was deduced from the reduced calcium concentration from day 14 to day 17 in the case of pure chitosan scaffolds in OS+ medium. The calcium phosphate precipitation responsible for this drop of calcium ion concentration may to be attributed primarily to the osteogenic differentiation additive, β-glycerophosphate, which leads to supersaturation of phosphate ions and favors mineral precipitation, as previously shown [22,23]. This influence is commonly attributed in osteoblast cell culture experiments to the start of mineralization, which is why in the current case of investigating the scaffolds influence on ion concentrations no cells were cultivated. This proves dystrophic, i.e., non-cell-controlled mineral precipitation caused by the scaffolds.

As expected, the mechanical characterization showed lower strength of the wet state scaffolds; it should be noted that this characteristic is important when referring to the stability of the specimens when used in a wet environment as a bone substitute material. It should also be noted that the mechanical properties of the scaffolds should be considered in light of load transfer at the cellular level rather than in the light of a load-bearing material, since the compressive strength of cancellous bone tends to have higher values than the presented scaffolds, in the range of about 150 kPa to 14 MPa [24]. The advantages of the chitin scaffolds could be demonstrated through the fact that the specimens did not show a loss of stability or physical degradation, as indicated by mechanical tests following their immersion in the cell culture medium. This finding corresponds to the observations of Freier et al. [25], who found only a slight decrease in mass (< 10%) for highly deacetylated chitosan even under addition of lysozyme to the liquid. In our study, the opposite effect was observed after incubation in which the stability of test specimens improved owing to mineral precipitation from the medium. The increase in strength was most marked for the more bioactive specimens (CA90/50-B and CA90/50-HA), as the mineral precipitation from the medium was significantly increased in these cases. Consequently, we may predict that the stability of scaffolds after implantation in a bone defect could be varied to a certain degree by manipulating the composition of the chitosan/mineral composites.

### 3.2. Influence of Scaffold Bioactivity on Osteoblast/Osteoclast Activity Ratio

The above-described influence of the mineral phases on the mechanical properties and bioactivity of the chitosan scaffolds will also be evident in the cellular reaction under mono- and co-culture conditions. The reduced proliferation of the hBMSCs over the first 14 days on CA90/50-B and CA90/50-HA, i.e., the specimens that caused the lowest calcium concentrations in response to the addition of the osteogenic medium confirmed the inherent influence of the biomaterial on the cells in culture. This inhibitory influence of reduced calcium ion concentration on hBMSCs was also shown in our previous investigations [26] and by Maeno et al. [12], who found an optimal calcium concentration in the range of 2–6 mM for culture of murine osteoblasts, and by Eklou-Kalonji et al. [27], who found an optimum at 3 mM calcium for porcine osteoblasts.

In addition to the effect of reduced calcium ion concentration in the medium, cell proliferation was further affected by the induced osteogenic differentiation of the hBMSCs. The differentiation of hBMSC, as reflected by the ALP maximum, was shifted to a later point in time for the highly bioactive specimens compared to the blank. The findings of a delay of ALP expression caused by the calcium phosphate particles accompanied by a decrease in calcium ion concentration in the hMSC culture media is in keeping with the results of a previous study [28].

Contrary to the assumption that the hMs or the osteoclasts are stimulated at lower calcium concentrations, as they show decreased activity under elevated extracellular calcium concentrations [29], the calcium-phosphate-free chitosan scaffold showed the highest TRAP5b activity of the cells. Moreover, the influence of the biomaterials on hBMSC proliferation and osteogenic differentiation became more marked in co-culture. It could be observed that hMs have a significantly lower LDH activity than hBMSCs in mono-culture, which was also seen in previous investigations [2]. Therefore, the increase in LDH activity in co-culture compared to hBMSC mono-culture can be attributed to the increased proliferation of hBMSCs and osteoblasts, respectively. This consideration is of course only valid if the LDH activity in co-culture is really only due to the hBMSC, and the mixed cultivation does not lead to increased LDH activity of the hMs or osteoclasts.

The calcium-phosphate-free reference showed the highest proliferation and the clearest osteogenic differentiation due to its comparatively small influence on the calcium concentration (permanently above 1.2 mM). These findings are in line with previous studies in which hBMSCs showed increased proliferation and differentiation in the range of 0.9–1.8 mM calcium in cell culture medium (on polystyrene, without a scaffold) [26]. The differentiation was only marginally prominent on calcium-phosphate-loaded scaffolds up to day 14. Furthermore it did not increase, as previously observed in mono-culture, since from day 14 no (either osteogenic or osteoclastic) additives were added to the medium during co-culture. Nevertheless, the co-culture of the hMs and osteoclasts together with the hBMSC/osteoblasts pre-cultured on the biomaterials shows, on the basis of TRAP5b expression, a clear maturation of osteoclasts. This finding becomes all the more important when we remember that in the hM mono-culture the artificially added additives (macrophage colony-stimulating factor (M-CSF) and RANKL) induced osteoclastic differentiation, whereas in co-culture, it was exclusively the cross-talk between the two cell types that caused the differentiation [2,30]. These results indicate that integration of the three different biomaterials into the natural remodeling process would be possible, to a certain extent, and that the physiological interaction of osteoblasts and osteoclasts is ensured. However, a shift of the osteoblast/osteoclast equilibrium towards osteoblasts, owing to the effect of the incorporation of commercial brushite and hydroxyapatite phases into the chitosan scaffolds and their influence on bioactivity does not seem possible.

### 3.3. Influence of Hemocyanin on Osteoblast/Osteoclast Activity Ratio

To provide an alternative biomaterial-based way to influence the cell balance in addition to the bioactivity of the materials, the cytocompatibility of chitosan scaffolds modified with the protein, hemocyanin, was investigated using different cell culture setups. It was confirmed that the proliferation of hBMSC could be significantly increased by the presence of hemocyanin proteins in the scaffold. This stimulating effect of the crustacean protein was previously observed after its immobilization on chitin scaffolds, which led to a significant increase in the adhesion and proliferation of hBMSCs [14]. The influence of proteins on osteoclasts was beyond the scope of this study.

In the present case, the protein modification of the chitosan scaffolds led to a significant increase in the cell number in mono-culture and even more so in co-culture. There was no influence of hemocyanin on ALP activity comparing osteoblasts in mono-culture on CA90/50 and CA90/50+Hemo. The influence of the hemocyanin on cell differentiation could be observed only in co-culture. The increase in osteoblast differentiation (ALP activity) occurred in co-culture, even though osteogenic additives were not used from day 14 onwards. This may be considered as proof of performance of hemocyanin in increasing the osteoblast/osteoclast activity ratio. However, in cultures, the effect of the hemocyanin proteins was primarily observed on osteoblast cell count (LDH) and differentiation in co-culture (ALP), since osteoclastic maturation (TRAP5b activity) showed equal trends with increased differentiation of co-cultured osteoclasts on CA90/50 only at 35 days/21 days. Since the approach of using crustacean proteins to modify chitosan bone substitute scaffolds to enhance their bone biocompatibility is rather new, no studies for a quantitative comparison are available. In general, the approach can be seen as belonging to the category of various publications on spongy scaffolds treated with pro-osteogenic proteins [31,32]. A possible explanation for the stimulative effect of hemocyanin is its role in both oxygen homeostasis and phenoloxidase activity (catalyze the hydroxylation of monophenols or the oxidation of o-diphenols to o-quinones, or both) [33]. The inhibition of differentiation markers in bone osteoblastic cells was shown by Mody et al., and furthermore, Marrazzo and O’Leary collected a comprehensive overview of studies demonstrating the advantageous properties provided by the incorporation of specific natural antioxidants into various biomaterials [34,35]. Thus, the hemocyanin that was immobilized on the scaffolds may indirectly influence the reactive oxygen species concentration and the oxidative stress balance in its vicinity in cell culture as well as in future application in the implantation site.

Finally, as a result of the cross-talk between osteoblasts and osteoclasts, an osteoblast-based increase in osteoclast maturation could be induced. Thus, it was shown that hemocyanin modification can promote osteoblastogenesis, which might be a future tool for directly promoting the healing of large bone defects or those in pre-diseased, e.g., osteoporotic, bones.

## 4. Materials and Methods

The first step in the study was the preparation of chitosan scaffolds that would be suitable for modification with both calcium phosphate minerals and crayfish hemocyanin. The modification was then performed either by incorporating the mineral phases during the scaffold fabrication process or by subsequent modification of the scaffolds with hemocyanin by dip-coating. The scaffolds were evaluated in terms of bioactivity (calcium ion binding and release, by incubation in physiological solutions) and mechanical compressive strengths (in the initial dry state and after incubation). Cytocompatibility was assessed by cell culture of hBMSC and their differentiation into osteoblasts and by cell culture of hM and their maturation to osteoclasts. In addition, co-culture of osteoblasts and hM was used to determine the influence of the scaffold material on the osteoblast/osteoclast activity ratio.

### 4.1. Preparation of Calcium-Phosphate-Modified Chitosan Scaffolds

Chitosan scaffolds were produced by lyophilization and stabilization with NaOH after adjustment of the method described previously [36,37]. Briefly, chitosan (Heppe Medical Chitosan HMC+) with a degree of deacetylation of 90% and a viscosity of 50 mPas was used; the material was thus designated CA90/50. The chitosan was dissolved as a 1 wt% solution in 2% acetic acid, and non-soluble components were removed by centrifugation. For modification, the calcium phosphate, either brushite (Sigma-Aldrich, Darmstadt, Germany) or hydroxyapatite (INNOTERE), was suspended in the chitosan solution in ratio of chitosan to calcium phosphate of 1/1 *wt*/*wt*). These specimens were designated CA90/50-B and CA90/50-HA, respectively.

Scaffolds for bioactivity and cytocompatibility tests were produced as follows: 1 mL of chitosan suspension was transferred into 48-well plates and then freeze-dried (Epsilon 2-4 LSC, CHRIST). Specimens for uniaxial compression tests were produced from 1.5 mL of chitosan suspension. To stabilize the pore structure, the chitosan scaffolds were soaked with 1 M NaOH (Fluka, München, Germany). Thereafter, depending on their size, the specimens were incubated in 1.0 mL or 1.5 mL of NaOH for 120 min. Specimens were then rinsed eight times for 10 min in deionized water. Modification of the specimens with hemocyanin was accomplished by dip-coating, as described in detail in Section 2.3. Finally, the specimens were lyophilized again and sterilized by gamma irradiation (25 kGy, Synergy Health).

### 4.2. Scaffold Characterization

#### 4.2.1. Bioactivity

The bioactivity of the cylindrical scaffolds (h = 5 mm, d = 11 mm) was analyzed as a function of time and of scaffold composition. For this purpose, three specimens each were incubated for 28 days in the following four different types of cell culture medium (α-MEM, Biochrom, Darmstadt, Germany). The basal medium, without osteogenic supplements (designated α-MEM OS-), was prepared with 10% fetal calf serum, 100 U/mL penicillin, 100 μg/mL streptomycin, and 2 mM l-glutamine. The supplemented medium (designated α-MEM OS+) was prepared by supplementing the basal medium with the osteogenic additives, ascorbic acid 2-phosphate (50 µM), dexamethasone (10 nM), and β-glycerophosphate (10 mM) (purchased from Sigma-Aldrich). In addition, a medium similar to the basal medium but without CaCl_2_ addition was purchased from Biochrom. In this medium (designated α-MEM low Ca), a low calcium concentration of about 0.4 mM was present owing to the natural amount of calcium in fetal calf serum. For co-culture, a regime of two different media was used (designated α-MEM OS co-culture). Incubation was performed for the first 14 days in α-MEM OS+ to stimulate the osteogenic differentiation of hBMSCs and from day 14 to day 28 in α-MEM OS- to allow osteoclast maturation.

#### 4.2.2. Mechanical Stability

For mechanical characterization of chitosan scaffolds, compression tests were carried out in both the dry and wet states, immediately after incubation in H_2_O during specimen preparation and after incubation in α-MEM OS- for 28 days. For each type of chitosan scaffold, 6 specimens were tested in a uniaxial compression test with a universal testing machine (Instron 5566, Norwood, UK). The test speed was 5 mm/min, with a test preload of 0.5 N. The test was carried out to a compression of 50%. Before the test, the height and diameter of the specimens were measured with a digital caliper.

### 4.3. Isolation of Hemocyanin from Crayfish Hemolymph and Scaffold Modification

Extraction of hemolymph and isolation of hemocyanin was performed, as previously described [14]. In brief, hemolymph was obtained by puncturing the base of the 5th walking legs of *C. quadricarinatus* pre-molt individuals at Ben-Gurion University of the Negev (Israel). Hemolymph was mixed with 7% EDTA, as an anti-coagulant, in 1:1 ratio and 2 mM phenylmethylsulfonyl fluoride (PMSF, Sigma-Aldrich) was added for protease inhibition. The diluted hemolymph was centrifuged at 1500 rcf for 15 min, and the supernatant was mixed with a saturated solution of sodium bromide. Hemocyanin was purified by ultracentrifugation at 10^5^ rcf for 48 h. The grey fraction was collected, dialyzed against phosphate-buffered saline (PBS), filtered, and lyophilized.

Scaffold modification with hemocyanin was performed by incubation of 36 scaffolds in an aqueous solution of 12.5 mg hemocyanin in 25 mL of double distilled water (0.5 mg/mL = 0.35 mg hemocyanin per each scaffold) at 4 °C. After incubation for 3 days, the specimens were gently washed in water. Thereafter, the specimens were lyophilized and then sterilized by gamma radiation.

### 4.4. Cell Culture Experiments

#### 4.4.1. Human Bone Marrow Stromal Cells

After informed consent had been obtained from the patients and approval had been granted by the local ethics committee (10.12.2004: EK263122004), hBMSCs were obtained from bone marrow aspirates at Medical Clinic I, University Hospital Carl Gustav Carus Dresden of the Technische Universität Dresden (Germany). Cells were expanded in Dulbecco’s modified Eagle’s medium (DMEM) containing 10% fetal calf serum, 1% penicillin-streptomycin, and 2 mM l-glutamine in a humidified 5% CO_2_ atmosphere at 37 °C and used for cell culture experiments in passage 5. The medium and all supplements were obtained from Biochrom. The scaffolds were pre-incubated in cell culture medium for 1 day in 48-well plates.

The cells were seeded at a density of 2.8×10^4^ cells (hBMSC) on the scaffolds cylinder top surface of 34.5 mm^2^ by drop-seeding (100 µL). Thereafter, the scaffolds were incubated in a humidified 5% CO_2_ atmosphere at 37 °C for 1 h. Cytotoxicity tests were started 1 h after seeding by adding α-MEM OS-. The medium was exchanged twice a week. After 3 days, cultivation of half the specimens with seeded cells was continued in α-MEM OS+ (50 µM ascorbic acid 2-phosphate, 10 nM dexamethasone, 10 mM β-glycerophosphate) to induce osteogenic differentiation. After 1, 14, 28, and 35 days, the specimens with the cells were rinsed in 1 mL of PBS at 37 °C and stored at −80 °C for later biochemical analysis of proliferation and differentiation (described below).

#### 4.4.2. Human Monocytes

hMs were isolated from buffy coats of healthy adult donors (German Red Cross), as described previously [2] using the OptiPrep^TM^ (ProGen Biotechnik, Heidelberg, Germany) density-gradient medium technique with some modifications. The monocyte-enriched peripheral blood mononuclear cell (PBMC) fraction was collected, washed with PBS containing 2 mM EDTA and 0.5% bovine serum albumin, and centrifuged at 400 rcf for 10 min. The hMs were purified via magnetic activated cell sorting by negative selection using Monocyte Isolation Kit II (Miltenyi, Bergisch Gladbach, Germany), according to the manufacturer’s instructions, and then counted. The hMs were seeded at a density of 3.0×10^5^ cells (hMs) by drop-seeding on chitosan scaffolds and cultivated with α-MEM supplemented with 7.5% fetal calf serum (heat inactivated), 7.5% human serum, and 1% penicillin-streptomycin (purchased from Biochrom). For hM mono-cultures, 25 ng/mL M-CSF (PeproTech, New Jersey, USA) was supplemented immediately upon starting the cultures [38]. After 2 days of cultivation, RANKL, 50 ng/mL, was added to induce osteoclast formation. Medium was exchanged twice a week.

#### 4.4.3. Co-Culture of hBMSC/Osteoblasts and hM/Osteoclasts

Chitosan scaffolds were pre-incubated for 24 h in basal medium in 48-well plates. The hBMSCs were seeded as for hBMSC mono-culture (2.8×10^4^ cells per scaffold). Osteogenic differentiation was induced from day 3. Over the first 14d, the cells were allowed to differentiate into osteoblasts. After first 14 days, the addition of hM was performed, which is being designated as 14d/1d, giving points of time of pre-cultured osteoblast and monocyte culture. The addition of hMs (3.2×10^5^ cells per scaffold) was accompanied by change of medium from α-MEM OS+ to MEM supplemented with 7.5% fetal calf serum (heat inactivated), 7.5% human serum, and 1% penicillin-streptomycin. It is noteworthy that neither osteoblastic nor osteoclastic supplements were added to the medium from the start of co-culture period (14d/1d) until its end. The cells were cultured until 35d/21d, while medium was exchanged twice a week.

### 4.5. Biochemical Analysis

Cellular lactate dehydrogenase (LDH) was used as a quantitative marker for cell number to study cellular adhesion and proliferation in vitro. Stored specimens (with cells on chitosan scaffolds) were thawed for 30 min on ice and afterwards incubated in 0.5 mL of lysis buffer (PBS with 1% Triton X-100) for 50 min on ice. A commercial LDH-Cytotoxicity Detection Kit (Takara, Kusatsu, Japan) was used to determine LDH activity. An aliquot of cell lysate was mixed with LDH substrate buffer, and the enzymatic reaction was stopped after 30 min with 0.5 M HCl. The absorbance was measured at 492 nm using a UV/Vis spectrometer (Tecan, Männedorf, Switzerland). The LDH activity was correlated with the cell number using a calibration line of cell lysates with a defined cell number [39]. To obtain a calibration curve 2 × 10^5^, 1.5 × 10^5^, 1 × 10^5^, 7.5 × 10^4^, 5 × 10^4^, 2.5 × 10^4^, 1 × 10^4^, and 0.5 × 10^4^ cells were seeded on polystyrene and frozen directly afterwards. The cells were lysed in the same way to allow calculation of cell number from LDH activity. Lysis buffer and scaffolds without cells were used as blanks.

#### 4.5.1. ALP Activity

ALP activity as a marker for osteogenic differentiation was determined spectroscopically at 405 nm using *p*-nitrophenyl phosphate (Sigma-Aldrich) as the substrate. Therefore, 125 µL of 1 mg/mL substrate solution (2 mg *p*-nitrophenyl phosphate in 0.1 M diethanolamine, 0.1% Triton X-100, and 1 mM MgCl_2_, pH 9.8) was added to 25 µL of lysate. After incubation for 30 min at 37 °C, the reaction was stopped with 50 µL of 1 M NaOH. Absorption was measured using a UV/Vis spectrometer (Tecan) at 405 nm. A calibration curve was constructed from different concentrations of *p*-nitrophenol (PNP). Lysis buffer and scaffolds without cells were used as blanks.

#### 4.5.2. TRAP 5b Activity

Osteoclast differentiation was evaluated by measurement of TRAP 5b activity according to a modified protocol based on Janckila et al. [40]. Cell lysates were added to TRAP 5b reaction buffer as a substrate consisting of 2.5 mM N-ASBI-P (Sigma-Aldrich) in 100 mM Na acetate (Sigma-Aldrich) buffer containing 50 mM Na tartrate (Sigma-Aldrich), 2% NP-40 (Sigma-Aldrich), and 1% ethylene glycol monomethyl ether (EGME, Sigma-Aldrich) adjusted to pH 6.1. The mixtures were incubated at 37 °C for 1 h. The enzymatic reaction was stopped by adding 0.1 M NaOH. Fluorescence was measured at an excitation wavelength of 405 nm and an emission wavelength of 535 nm. The relative fluorescence units were correlated to a TRAP 5b standard.

### 4.6. Statistics

All measurements were performed at least in triplicate (mechanical tests with 6 specimens) and results are expressed as means ± standard deviation. One-way or two-way analysis of variance (ANOVA) with Bonferroni post hoc test were applied for statistical analysis where applicable, and *p* values < 0.05 were considered significant and indicated by an asterisk. To avoid the type II error, the statistical analyses were conducted only across groups and only in selected cases, such as the cell number development on day 35 between hBMSC and co-culture or TRAP 5b as a differentiation marker also on day 35 between monocytes and co-culture.

## 5. Conclusions

In this study, two types of effects influencing osteoblast/osteoclast equilibrium in co-culture over chitosan scaffolds were investigated. The first material modification was performed by the incorporation of calcium phosphate (in the form of brushite or hydroxyapatite) into the chitosan scaffolds. These mineral phases acted as nucleation germs for calcium phosphate precipitation, which was shown by a decreased calcium concentration in the medium. Mineral incorporation and precipitation are associated with a trend towards increased strength of the scaffolds. Furthermore, the cells on the mineral-containing scaffolds reacted to the non-physiological lowered calcium ion concentrations in mono-culture and co-culture with reduced adhesion and reduced or delayed osteoblast differentiation and osteoclast maturation.

The second material modification by immobilization of hemocyanin proteins on the chitosan scaffolds resulted in significantly increased proliferation of hBMSCs and equivalent osteoclast maturation compared to the blank in mono-cultures. Co-culture studies showed the stimulatory effects of hemocyanin-modified scaffolds on both hBMSC count and osteoblastic differentiation compared to the blank scaffolds. This was followed by differentiation of hMs to osteoclasts without the need for any additional osteoclastic supplements. This finding confirms that in the future it might be possible to specifically support bone healing by influencing osteoblasts and osteoclasts by using specially designed materials with particular inherent properties for bone regeneration.

## Supplementary Materials

The following are available online, Figure S1: Scanning electron micrographs of plain as well as brushite and hydroxyapatite modified chitosan scaffolds, respectively. Secondary electron images were taken at 3 keV with an XL30ESEM-FEG (FEI).

## Abbreviations

ALP	alkaline phosphatase
α-MEM OS-	alpha minimum essential medium without osteogenic supplements
α-MEM OS+	alpha minimum essential medium with osteogenic supplements
CA90/50	scaffolds from chitosan with a degree of deacetylation of 90% and a viscosity of 50 mPas
CA90/50-B	chitosan scaffolds with brushite
CA90/50-HA	chitosan scaffolds with hydroxyapatite
DMEM	Dulbecco’s modified Eagle’s medium
EDTA	ethylenediaminetetraacetic acid
hBMSC	human bone marrow stromal cells
hM	human monocytes
LDH	lactate dehydrogenase
M-CSF	macrophage colony-stimulating factor
N-ASBI-P	naphthol AS-BI-phosphate
PBMC	peripheral blood mononuclear cell
PBS	phosphate-buffered saline
PMSF	phenylmethylsulfonyl fluoride
PNP	p-nitrophenol
RANKL	receptor activator of NF-κB ligand
TRAP	tartrate-resistant acid phosphatase
