# Supplementary material for: Hemocyanin Modification of Chitosan Scaffolds with Calcium Phosphate Phases Increase the Osteoblast/Osteoclast Activity Ratio—A Co-Culture Study
Source: Molecules. 2020 Oct 7;25(19):4580. doi: 10.3390/molecules25194580 (PMC7582980; doi:10.3390/molecules25194580)
Supplement: Supplementary file 1 [file molecules-25-04580-s001.pdf]

Supplementary

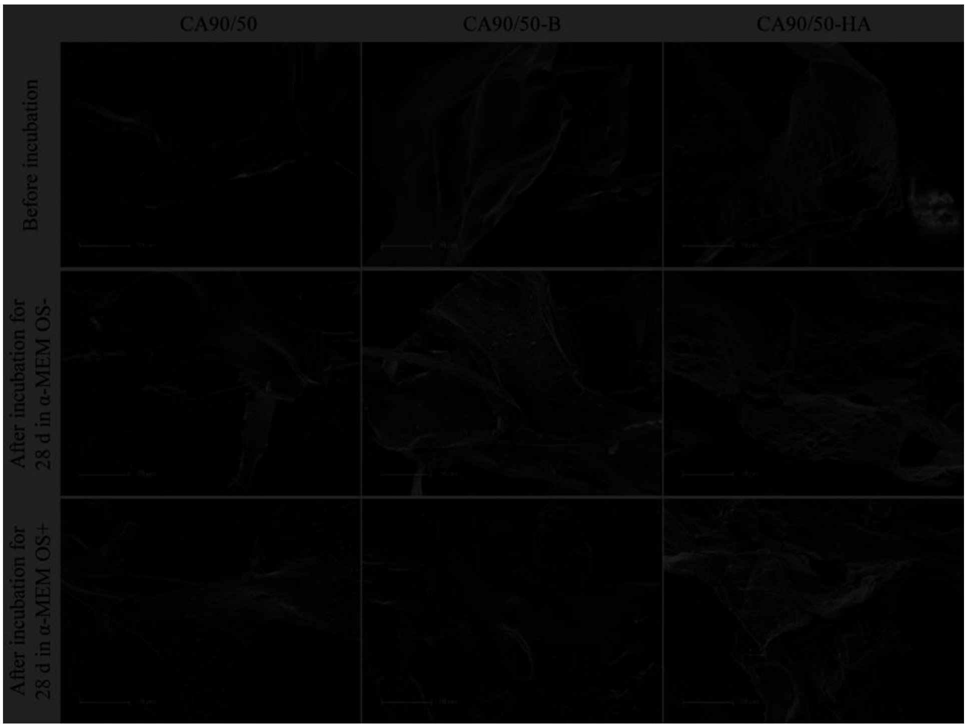

Figure S1 Scanning electron micrographs of plain as well as brushite and hydroxyapatite modified chitosan scaffolds, respectively. Secondary electron images were taken at 3 keV with an XL30ESEM-FEG (FEI).
